# Stabilization of macular, peripapillary and papillary vascular parameters after XEN and trabeculectomy visualized by the optical coherence tomography angiography

**DOI:** 10.1038/s41598-022-22091-6

**Published:** 2022-10-14

**Authors:** Emanuel Reitemeyer, Milena Pahlitzsch, Anna Cornelius, Daniel Pilger, Sibylle Winterhalter, Anna-Karina B. Maier

**Affiliations:** Department of Ophthalmology, Campus Virchow- Klinikum, Charité—Universitätsmedizin Berlin, corporate member of Freie Universität Berlin, Humboldt- Universität Zu Berlin and Berlin Institute of Health, Augustenburger Platz 1, 13353 Berlin, Germany

**Keywords:** Medical research, Outcomes research

## Abstract

To analyze the effect of filtration in glaucoma surgery, XEN versus trabeculectomy, on the vessel area density (VAD) of the macular, papillary and peripapillary regions using optical coherence tomography angiography (OCT-A). This prospective cohort study analyzes the vascular architecture of 47 eyes of 45 patients after two different filtrating surgery procedures (XEN stent and trabeculectomy (TE)) using the OCT-A. Participants who had an outsourced medical anti-glaucoma therapy received filtrating surgery in a 2:1 (XEN: TE) ratio. The primary outcome measurements were changes in the VAD in various layers of retinal perfusion and the foveal avascular zone (FAZ). Both interventions achieved a significant postoperative reduction in IOP (XEN 17.6 ± 3.8–13.7 ± 3.8 mmHg; TE 21.2 ± 5.4–8.8 ± 2.6 mmHg). VAD values did not change significantly after filtrating surgery. Comparing both procedures, a significantly higher VAD for patients treated with TE was seen for the superficial vessel complex (SVC) 375 µm and 750 µm (p = 0.011, p = 0.017), deep vessel complex (DVC) 375 µm (p = 0.029) and the optic disc (p = 0.028) after 6 months, while all other parameters did not differ significantly. In conclusion, VAD does not significantly improve after filtrating surgery in preoperative moderately IOP elevated eyes. The IOP lowering effect of filtrating surgery, however, can stabilize vascular parameters in all layers of perfusion.

## Introduction

Glaucoma is one of the leading causes of vision impairment and accounts for about 3.6 million of the 33.6 million adults, who are aged 50 and above, living with blindness in 2020^1^. In addition to intraocular pressure (IOP)-dependent mechanisms, vascular mechanisms such as in the context of diabetes or arterial hypertension, play a role in the pathology of glaucoma and influence the course of the disease as observed in systematic reviews^2^ or clinical studies like AGIS^3^. Aside from an elevation or change in IOP, Yarmohammadi et al.^4^ provide evidence that changes in retinal vessels might also be relevant and have even been detected prior to visual field damage.

A new approach to investigate the effects of vascular dysregulation and their relations is the analysis of ocular perfusion using the optical coherence tomography angiography (OCT-A). The analysis of microvasculature changes might be a useful biomarker for detecting vascular insufficiency—known to play a key role in glaucomatous changes of the eye^2,3,5,6^. Additionally, monitoring the vessel architecture might be a major factor in the decision-making of surgical glaucoma therapy. In previous studies using this new approach, patients diagnosed with glaucoma, have been associated with a lower vessel area density (VAD) compared to healthy persons^7–9^.

The reduction of IOP by medical treatment or by surgical intervention is currently the only effective known modality to prevent further optic disc damage^10,11^. Filtrating surgeries show the strongest effect of IOP lowering interventions^12^, with trabeculectomy (TE) still being the standard surgical therapy. In recent years, however, new glaucoma devices have evolved, for example the XEN stent (Allergan Inc., CA, USA). The subconjunctival filtration via XEN stent shows good IOP lowering effects with a better safety profile^13–17^. Although first studies showed a better IOP lowering effect by trabeculectomy compared to XEN stent, it proved a higher risk for postoperative hypotony^18–20^.

The effect of IOP lowering by trabeculectomy on the VAD by OCT-A show controversial findings regarding the influence of IOP-lowering surgery on the retinal and (peri) papillary perfusion. While some studies show an increase in optic disc perfusion^21^ or peripapillary perfusion^22–24^, other studies do not^25,26^.

Because of differences of the IOP lowering effect in the early and later postoperative period between the filtrating procedures—trabeculectomy and XEN stent—and its possible effects on the vessel area density, we investigated changes in VAD in the macular, peripapillary and papillary layers in glaucomatous eyes after IOP lowering by trabeculectomy and by XEN stent using OCT-A.

## Patients and methods

### Study setting

In this prospective, comparative, outcome study, we included eyes from patients that underwent filtrating glaucoma surgery. This included either minimally invasive glaucoma surgery (MIGS) representative XEN stent implantation or standard trabeculectomy at the Department of Ophthalmology, Charité—Universitätsmedizin Berlin. Patients were recruited and their data recorded from 08/2018 to 06/2021. This trial was approved by the ethics committee, Charité—Universitätsmedizin Berlin, EA4/168/17. This study adhered to the ethical standards of the Declaration of Helsinki. All patients involved in this study provided written, informed consent for participation in the study.

### Trial population

Patients were eligible if they were at least 18 years of age and had a diagnosed form of glaucoma including open-angle glaucoma (primary and pseudoexfoliative glaucoma), pigmentary glaucoma, normal tension glaucoma and angle-closure glaucoma. The key indication for patients to undergo surgery was the progression of glaucomatous damage of the optic disc, despite maximum and outsourced anti-glaucomatous medical treatment or an individually assessed intraocular pressure that would be associated with a predicted damage of the optic disc or visual field. Key exclusion criteria were glaucoma patients who received a different treatment, patients with difficult admissions findings like extreme myopia, cataract, uveitis, a lack of compliance or unusable preoperative OCT-A pictures.

After surgery follow-ups were scheduled as follows: 3 days, 6 weeks, 3 months and 6–12 months (average 7.3 ± 2.3 months). The following parameters were collected on any follow-up appointment: blood pressure, pulse rate, number of anti-glaucomatous medication, IOP (using Goldmann-applanation-tonometry), BCVA and OCT (-A) images via device examination on the eye that underwent surgery. Additionally, any complications and additional treatments associated with the surgery were recorded.

Patients received surgical treatment, XEN stent implantation or trabeculectomy according to the recommendation by the European Glaucoma Society. Stage of glaucoma, IOP before treatment, age and life expectancy, rate of progression and presence of risk factors for progression were used for defining the individual target pressure.

### Primary and secondary outcomes

The primary outcome was a composition of changes in the VAD in the macula, the optic disc and the peripapillary regions. In addition to the VAD, changes in the retinal nerve fiber layer (RNFL) and of the foveal avascular zone (FAZ) were also seen as primary outcomes. Secondary outcomes were changes in IOP, changes in sight, and changes in number of anti-glaucoma medication.

#### Primary outcome measurements

To record both the OCT and the OCT-A images, the Heidelberg Engineering Spectralis (Heidelberg Engineering GmBh, Heidelberg, Germany) was used. It primarily detects intravascular motion of corpuscular blood components and hence, indirectly allows for a depiction of intraocular vessels.

Most images were aimed to be taken in a 15 × 15 window and the image quality range was set between 20 and 50.

##### Vessel area density

All calculations regarding the VAD were done with the ImageJ software program (publicly available and provided by the National Institutes of Health, Bethesda, MD, USA)^27^.

1. OCT-A images were imported into ImageJ^27^ and converted into 8bit images.
2. The margins for the optic disc were determined using the scales set outside the en-face image. The scales have been determined prior the import using the associated OCT b-scans. The optic disc was defined anatomically by the absence of the bruch membrane.
3. The total area of the optic disc was circled using the oval selection tool and its area calculated and measured as the total pixel count (TPC).
4. The image was then binarized using the Niblack’s method^21,28^ with a threshold set to 255. The obtained black and white were also measured for their area as the white pixel count (WPC) value. The WPC was divided by the TPC. The number deducted from the results therefore gave a percentage area of white particles (i.e. vessels)/VAD.
5. The VAD value was calculated for the following regions:
6. Macular, optic disc, peripapillary superficial vascular complex (SVC), peripapillary deep vascular complex (DVC), peripapillary choriocapillary layer (CCL), peripapillary chorioideal layer (CL).
7. The peripapillary regions were defined as the circular region around the optic disc with a distance of 375 µm and/or 750 µm to the optic disc. Some OCT-A images were not suitable for a 750 µm analysis because of artifacts or a lack of appropriate size.

**Figure 1:**
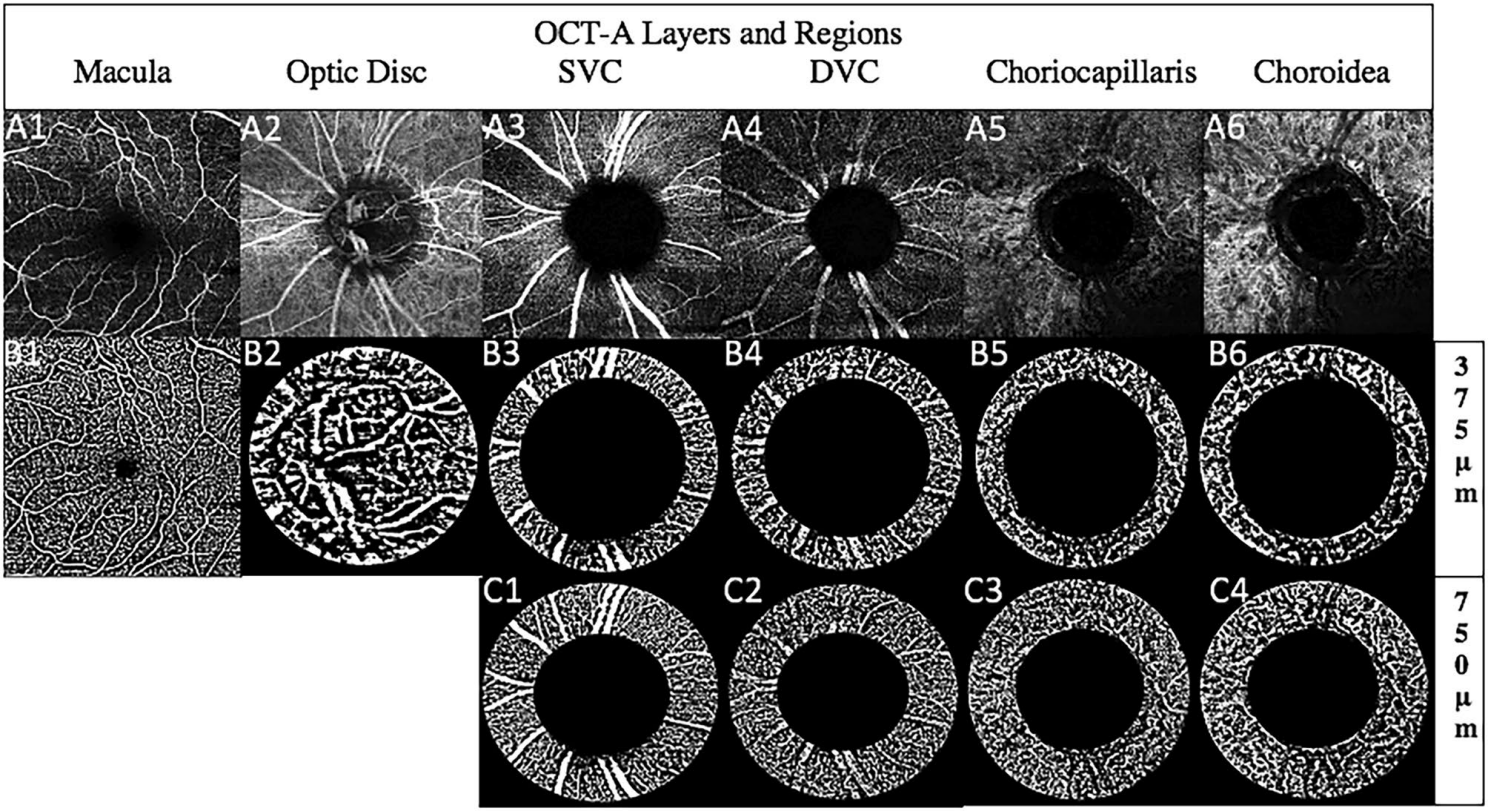
Shows examples of OCT-A en-face images. **A**(**1–6**) = Original OCT-A macular A1, optic disc A2 and peripapillary layer including SVC A3, DVC A4, CCL A5 and CL A6. **B**(**1–6**) = equivalent binarized OCT-A images of the original images (B3-6, 375 μm peripapillary circle and C1-4, 750 μm peripapillary circle).

Figure 1 offers a visualization of a complete set of OCT-A images per patient per examination date.

##### Foveal avascular zone

All measurements regarding the FAZ were done using the tracing tool on the Heidelberg Engineering Spectralis device on the original images. The avascular zone was defined as the zone in which no white pixel signal was detected. To minimize the bias and examiners subjective tracing method, each FAZ was drawn by two experienced conductors (ER, AC) separately.

**Figure 2:**
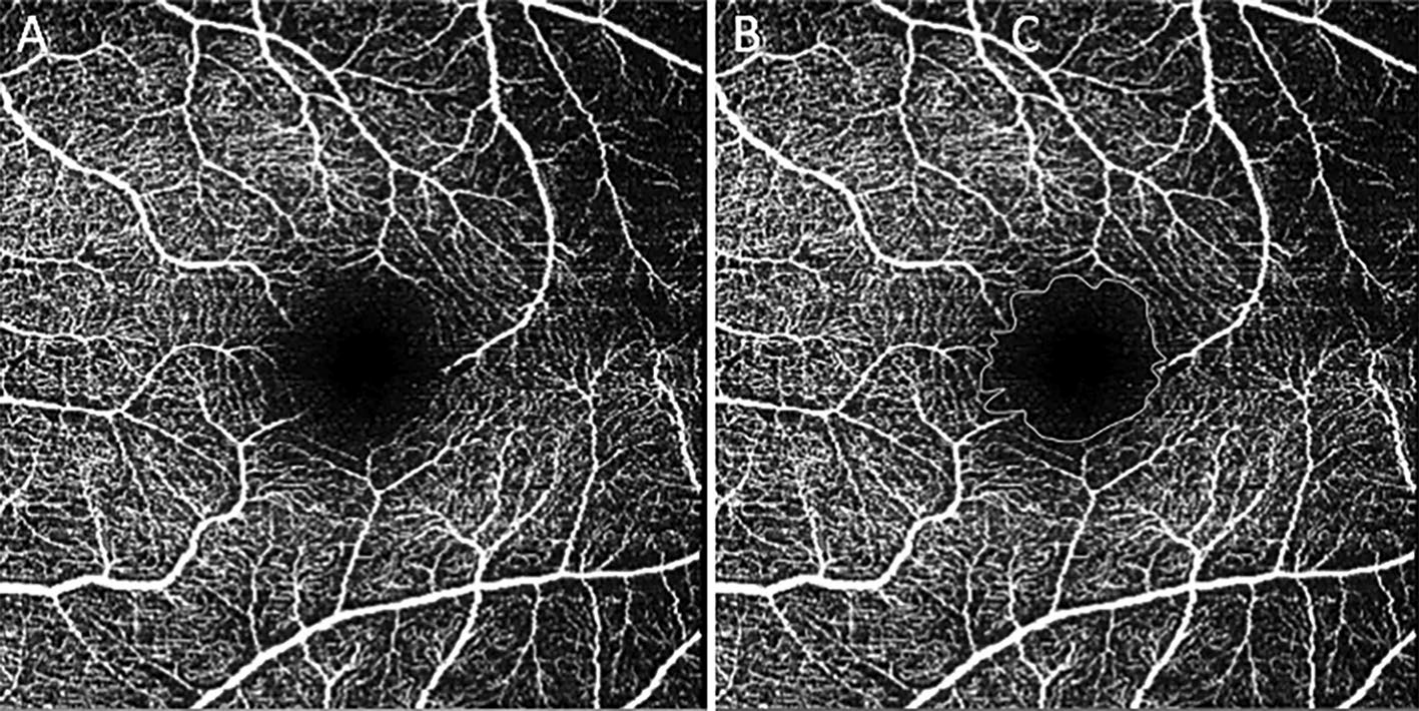
OCT-A images of the macula with the FAZ in the center showing the original (**A**) and the traced FAZ (**B**).

An exemplary FAZ tracing is shown in Fig. 2.

##### Retinal nerve fiber layer (RNFL)

The RNFL values were obtained for the peripapillary region and defined as the area between the internal limiting membrane and the ganglion cell layer. The RNFL values were divided into the following sections: superior, inferior, nasal, temporal and average.

##### Retinal thickness

The retinal thickness values were obtained for the macular region. The retinal thickness values were divided into the following sections: superior, inferior, nasal, temporal and average.

### Statistical methods

Statistical analysis was performed using IBM SPSS Statistics program (IBM Corp. Released 2019. IBM SPSS Statistics for Windows, Version 26.0. Armonk, NY: IBM Corp). A sample size calculation was based on the assumption of a mean VAD SVC 32.97% ± 1.11% based on the available data of our study group using ImageJ^27^ analysis and a distribution of 2:1^28^. At a power of 80% and an alpha level of 5%, we estimated that a group size of 36 patients would allow detecting a difference of 1.0% VAD. Descriptive statistics were expressed as mean ± standard deviation (SD). Normality was tested for all outcome measures with the Shapiro-Wilktest and the appropriate statistical test was used. A correlation analysis between RNFL thickness, mean deviation, vascular risk factors and VAD; as well as reduction in intraocular pressure and changes in VAD; was performed using the Spearman and Pearson test. Differences were considered statistically significant when p-values were less than 0.05.

## Results

Of 60 patients initially assessed for eligibility, a total of 13 TE patients and 32 XEN stent patients were enrolled in the study. Eight patients had to be excluded because of lost of follow-up, two patients because of poor image quality, one patient because of XEN stent removal within the first month, and four patients because of a secondary glaucoma due to a diagnosed uveitis.

Images had to be excluded or could not be analyzed for further analysis when image quality was too poor (below a signal strength index of 18) or if a patient missed the OCT-A imaging. Furthermore, the last follow-up appointment was extended to 6–12 months after filtrating surgery because of the COVID-19 pandemic. In total, data of 13 eyes of 13 TE patients and 34 eyes of 32 XEN stent patients were included because they reached the last scheduled follow-up.

Baseline characteristics of both treatment groups are demonstrated by Table 1. There were no significant differences between the two treatment groups regarding demographic characteristics and cardiovascular parameters (p > 0.05).

**Table 1:** Clinical characteristics of the patients at baseline.

Clinical parameters	XEN stent	TE	p-value
Number of patients	32	13	
Number of eyes	34	13	
Female sex in %	68	39	0.086^a^
Mean age in years (± SD)	74 ± 12	66 ± 18	0.198^b^
Pseudophakic IOL—in %	76	77	0.947^a^
Diabetes mellitus—in %	9	0	0.268^a^
Arterial hypertension—in %	47	39	0.596^a^
RRsystolic in mmHg (± SD)	141 ± 25	135 ± 2	0.526^c^
RRdiastolic in mmHg (± SD)	85 ± 12	80 ± 7	0.088^b^
MAP in mmHg (± SD)^¶^	104 ± 15	98 ± 10	0.319^b^
Pulse in n/min (± SD)	72 ± 8	71 ± 8	0.639^c^
Anti-glaucoma eye drops—no. (± SD)	3.2 ± 1.0	2.8 ± 1.5	0.420^b^

*IOL* intraocular lens, *RR* Riva-Rocci blood pressure, *MAP* mean arterial pressure.

^¶^The mean arterial pressure is the diastolic pressure added to one third of the difference between systolic and diastolic pressure in mmHg.

^a^Chi-Quadrat test.

^b^Mann-Whitney *U* test.

^c^*t* test.

Table 2 summarizes the clinical characteristics and diagnostic measurements of each group. There were no significant differences between the two groups regarding visual acuity, perimetrical mean deviation, RNFL or cup-to-disc ratio (CDR). However, the initial intraocular pressure was significantly lower in eyes receiving XEN stent surgery 17.6 ± 3.8 mmHg than TE 21.2 ± 5.4 mmHg (p = 0.013).

**Table 2:** Diagnostic measurements.

Parameters	XEN stent	TE	p-value
Visus in LogMAR (± SD)	0.27 ± 0.28	0.24 ± 0.30	0.745^b^
IOP in mmHg (± SD)	17.6 ± 3.8	21.2 ± 5.4	**0.013**^c^
VF MD in dB (± SD)	12.8 ± 6.6	9.4 ± 6.0	0.135^c^
CDR (± SD)	0.8 ± 0.2	0.8 ± 0.1	0.230^b^
**RNFL thickness in μm** (± SD)			
Average	63.3 ± 20.6	67.9 ± 14.8	0.474^c^
Superior	69.4 ± 25.5	83.1 ± 33.4	0.138^c^
Inferior	76.3 ± 30.9	79.9 ± 25.4	0.712^c^
Temporal	54.8 ± 19.7	58.2 ± 11.9	0.475^c^
Nasal	53.9 ± 20.4	51.4 ± 14.1	0.688^c^
SSI	23.7 ± 5.6	24.9 ± 6.3	0.399^b^
**Retinal thickness in μm** (± SD)			
Average	314.7 ± 40.0	308.9 ± 29.0	0.644^c^
Superior	314.8 ± 34.0	314.9 ± 27.5	0.991^c^
Inferior	313.1 ± 49.9	311.7 ± 27.3	0.925^c^
Temporal	304.0 ± 35.9	306.4 ± 20.5	0.826^c^
Nasal	326.0 ± 48.0	317.7 ± 31.1	0.568^c^
SSI	30.1 ± 4.0	27.8 ± 4.3	0.093^c^

Significant values are in bold.

Differences between the groups are shown as mean ± standard deviation.

*IOP* intraocular pressure, *VF MD* visual field mean deviation, *RNFL* Retinal nerve fiber layer thickness, *CDR* cup-to-disc-ratio, *pf* parafoveal, *SSI* signal strength index.

^a^Chi-Quadrat test.

^b^Mann-Whitney *U* test.

^c^*t* test.

Table 3 shows the angiographic parameters. Angiographic parameters for the macular and optic disc region did not differ significantly between the two groups. In the peripapillary region, the only statistically significant difference was measured for the CL in both the 375 µm (36 ± 4% versus 40 ± 5%, p = 0.013) and 750 µm diameter (36 ± 3% versus 38 ± 3%, p = 0.028) with lower VAD values for the patients that received a XEN stent.

**Table 3:** OCT-Angiography parameters.

OCT-A parameters	XEN stent	TE	p-value
**Macular OCT-A**			
FAZ in mm^2^	0.57 ± 0.29	0.59 ± 0.30	0.781^b^
VAD in %	29.0 ± 2.2	30.3 ± 3.6	0.163^c^
SSI	30.5 ± 3.7	30.7 ± 4.3	0.918^c^
**Optic disc OCT-A**			
VAD in %	36.3 ± 1.7	37.1 ± 2.4	0.206^c^
**Peripapillary OCT-A**			
VAD SVC 375 µm in %	37.0 ± 6.3	40.1 ± 5.5	0.123^c^
VAD DVC 375 µm in %	33.5 ± 3.0	35.0 ± 2.6	0.117^c^
VAD CCL 375 µm in %	34.0 ± 4.6	37.3 ± 5.4	0.050^c^
VAD CL 375 µm in %	36.0 ± 4.3	39.7 ± 4.7	**0.013**^**c**^
VAD SVC 750 µm in %	36.1 ± 5.3	38.8 ± 5.5	0.157^c^
VAD DVC 750 µm in %	31.7 ± 2.5	32.6 ± 2.1	0.267^c^
VAD CCL 750 µm in %	33.9 ± 3.1	35.5 ± 4.1	0.178^c^
VAD CL 750 µm in %	35.7 ± 3.0	38.1 ± 2.6	**0.028**^**c**^
SSI	31.1 ± 3.7	30.1 ± 2.6	0.444^c^

Significant values are in bold.

Differences between the groups are shown as mean ± standard deviation.

*FAZ* foveal avascular zone, *VAD* vessel area density, *SSI* Signal Strength Index, *SVC* superficial vascular complex, *DVC* deep vascular complex, *CCL* choriocapillaris layer, *CL* chorioideal layer, 375 µm radius around optic disc, 750 µm radius around optic disc.

^a^Chi-Quadrat test.

^b^Mann-Whitney *U* test.

^c^*t* test.

**Figure 3:**
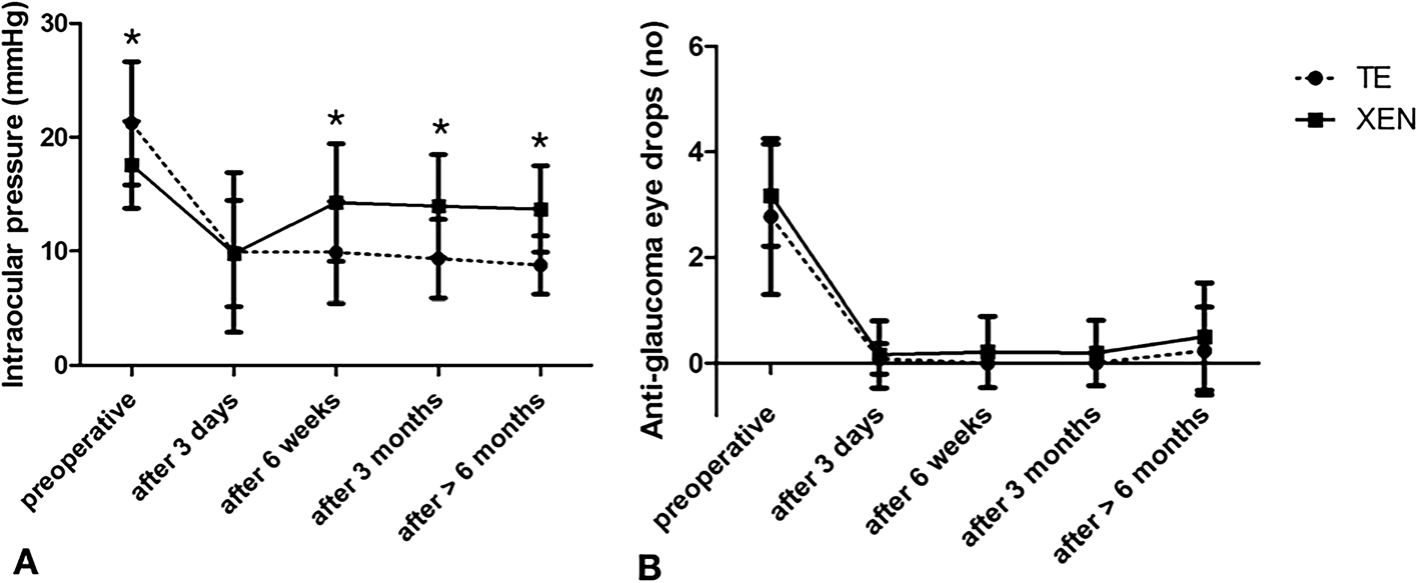
Change in (**A**) intraocular pressure (in mmHg) and (**B**) number of anti-glaucoma eye drops for the follow-up intervals. *marked significant differences between both treatment groups.

**Figure 4:**
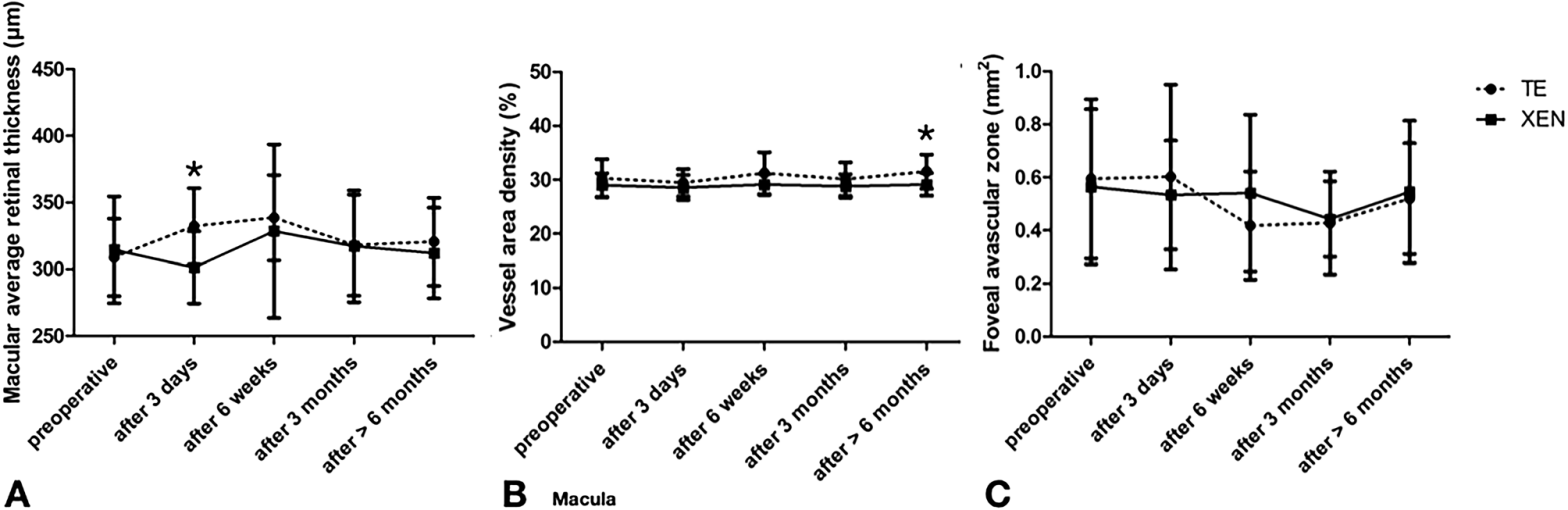
Change in (**A**) average retinal thickness (in µm), (**B**) macular vessel area density (in percentage) and (**C**) foveal avascular zone (in mm^2^) for the follow-up intervals. *marked significant differences between both treatment groups.

**Figure 5:**
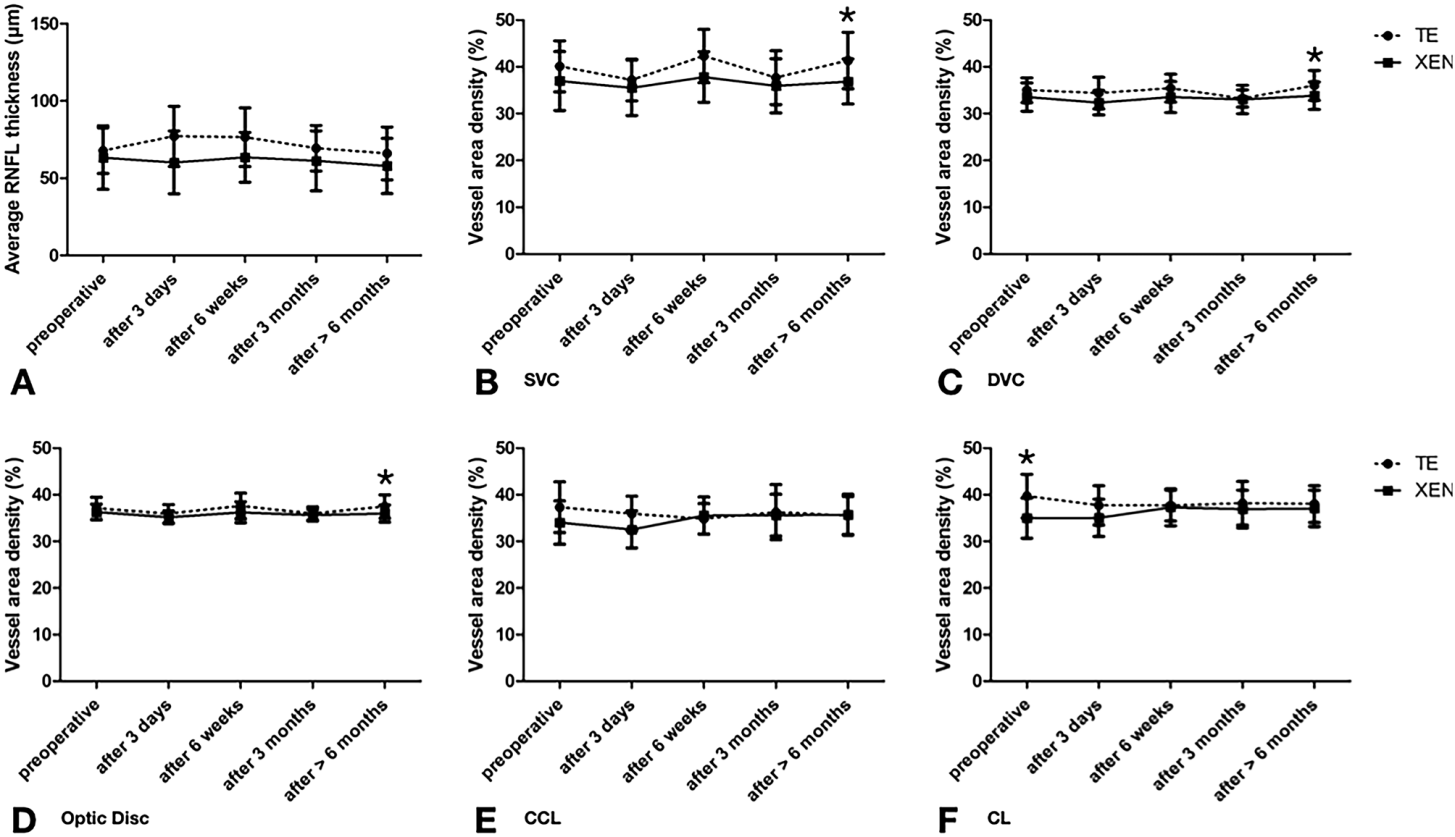
Change in (**A**) average RNFL thickness (in µm), (**B**) SVC superficial vascular complex (in percentage), (**C**) DVC deep vascular complex (in percentage), (**D**) optic disc (in percentage), (**E**) CCL choriocapillaris layer (in percentage) and (**F**) CL chorioideal layer (in percentage) for the follow-up intervals. *marked significant differences between both treatment groups.

Figures 3, 4, 5 show the course of the analyzed parameters over time. IOP and number of glaucoma medication decreased significantly for both treatment groups (XEN: p < 0.001, p < 0.001, TE: p = 0.001, p = 0.002) after more than 6 months compared to preoperative values (Fig. 3). Patients receiving a XEN stent had significantly higher IOP values post 6 weeks: p = 0.022, post 3 months: p < 0.001, post > 6 months: p < 0.001 (Fig. 3).

Analysis of the macular parameters showed no significant differences over time for both treatment groups (Fig. 4), but there was a significant difference between the two groups with a statistically significant higher macular VAD for patients receiving a TE treatment after 6 months (p = 0.008) (Fig. 4). Additionally, TE treated patients showed a significantly higher retinal thickness 3 days after surgery (p = 0.018), but also a significant better image quality (p = 0.028) compared to XEN treated patients. There were no further statistically significant differences regarding the parameters of the macula (Fig. 4).

Analyzing the peripapillary and optic disc area, RNFL decreased (p = 0.013) and VAD of CCL 375 µm increased (p = 0.012) significantly over time for eyes treated with XEN stent while all other parameters (peri-/papillary) showed no significant differences over time for both treatment groups (Fig. 5). Comparing both treatment groups, patients treated with TE showed significantly higher results in VAD of SVC 375 µm and 750 µm (p = 0.011, p = 0.017), in VAD DVC 375 µm (p = 0.029) and optic disc VAD (p = 0.028) after 6 months while all other peripapillary parameters did not differ significantly between the groups (Fig. 5).

The Spearman and Pearson correlation analysis (Table 4) were used to examine the correlations between OCT-A parameters and the structural parameter RNFL and the functional visual field mean deviation (MD). Regarding the preoperative data, MD and average RNFL both showed significant correlation for the macular VAD (r = − 0.573, p < 0.001, r = 0.329, p = 0.031 respectively) and the superficial vessel complex VAD (r = − 0.634, p < 0.001, r = 0.415, p = 0.004 respectively). MD additionally showed significant correlation to the optic disc VAD (r = − 0.326, p = 0.043) and the DVC VAD (r = − 0.402, p = 0.011).

**Table 4:** Correlation analysis.

Clininical parameters	Macular VAD	FAZ	Optic disc VAD	SVC VAD	DVC VAD	CCL VAD	CL VAD
**Preoperative**							
MD	− 0.573	0.189	− 0.326	− 0.634	− 0.402	− 0.001	− 0.016
	**p < 0.001**^b^	p = 0.277^a^	**p = 0.043**^b^	**p < 0.001**^b^	**p = 0.011**^b^	p = 0.995^b^	p = 0.923^b^
RNFL	0.329	0.076	0.008	0.415	− 0.099	− 0.266	− 0.220
	**p = 0.031**^b^	p = 0.627^a^	p = 0.956^b^	**p = 0.004**^b^	p = 0.514^b^	p = 0.074^b^	p = 0.142^b^
**After > 6 months**							
MD	− 0.667	0.237	− 0.577	− 0.642	− 0.460	0.115	0.160
	**p < 0.001**^b^	p = 0.191^b^	**p < 0.001**^b^	**p < 0.001**^b^	**p = 0.003**^b^	p = 0.485^b^	p = 0.924^b^
RNFL	0.498	− 0.039	0.550	0.550	0.258	− 0.418	− 0.309
	**p = 0.001**^b^	p = 0.809^b^	p < 0.001^b^	**p < 0.001**^b^	p = 0.080^b^	**p = 0.003**^b^	**p = 0.034**^**b**^

Significant values are in bold.

*MD* mean deviation, *RNFL* retinal nerve fiber layer thickness, *VAD* vessel area density, *FAZ* foveal avascular zone, *SVC* superficial vessel complex *DVC* deep vessel complex, *CCL* choriocapillaris layer, *CL* Chorioideal layer.

^a^Spearman.

^b^Pearson.

All values are shown as correlation coefficient and p value.

After 6 months, a similar pattern of statistically significant correlation can be observed. MD and average RNFL both showed significant correlation for the macular VAD (r, − 0.667, p < 0.001, r = 0.498, p = 0.001 respectively) and the SVC VAD (r = − 0.642, p =  < 0.001, r = 0.550, p < 0.001 respectively). MD additionally showed significant correlation to the optic disc VAD (r = − 0.577, p < 0.001) and the DVC VAD (r = − 0.460, p = 0.003) and average RNFL to the CCL (r = − 0.372, p = 0.007) and the CL (r = − 0.309, p = 0.034).

Further analysis was carried out using Spearman and Pearson to correlate reduction of IOP with change in VAD. All vessel complexes did not significantly correlate to the change in IOP as represented by Table 5.

**Table 5:** Correlation analysis.

Clinical parameters	Macular VAD after > 6 months—macular VAD preoperative	FAZ after > 6 months—FAZ preoperative	Optic disc VAD after > 6 months—optic disc VAD preoperative	SVC VAD after > 6 months—SVC VAD preoperative
IOP after > 6 months—IOP preoperative	− 0.235	0.282	− 0.184	− 0.131
	p = 0.145^b^	p = 0.078^a^	p = 0.216^b^	p = 0.384^b^

*VAD* vessel area density, *FAZ* foveal avascular zone, *SVC* superficial vessel complex.

^a^Spearman.

^b^Pearson.

### Post-surgery complications

Post-surgery 18 of 34 XEN stent eyes (53%) and 1 of 13 TE eyes (8%) received a bleb needling. 3 of 34 XEN stent eyes (9%) and 1 of 13 TE eyes (8%) were diagnosed with postoperative hypotony (< 6 mmHg).

## Discussion

IOP-lowering filtrating surgery (either XEN stent or TE) did not significantly change the vascular parameters of the papillary, peripapillary and macular region in preoperative moderately IOP elevated eyes. Comparing both treatments, TE treated patients showed minimally significant higher results in VAD (SVC, DVC and optic disc) than the XEN stent group after 6 months.

Both filtrating surgeries, XEN stent and TE, reduce the IOP significantly^12,14–17^. Similar to previous findings, the decrease in number of anti-glaucoma eye drops was comparable between both procedures, but the IOP-lowering effect was stronger in the TE group than in the XEN stent group^14–17^.

To our knowledge, this is the first report that analyzed VAD comprehensively by exploring the SVC, DVC, CL, CCL and optic disc layer in filtrating glaucoma surgery and thus advances glaucoma therapy concepts. Analyzing the influence of IOP lowering on the microvasculature, we found no significant changes in VAD over time in all regions of perfusion—except for the CCL layer in XEN stent patients. Comparable to these results, Zéboulon et al.^29^ did not detect any changes in the VAD of the superficial peripapillary layer 1 month after TE. With similar results 6 months post-surgery, Lommatzsch et al.^25^ also did not detect changes in the papillary and peripapillary layer. However, a significant increase in VAD of the peripapillary layer following 3 months after TE surgery was reported by Shin et al.^23^, In et al.^24^ and Miraftabi et al.^30^. Kim et al.^21^ could detect papillary VAD increase 3 months following TE but not in the peripapillary layer.

Considering these irregular post-surgery follow-up intervals found in current literature, we deducted that the time period selected to detect changes in VAD could be crucial because of influential factors like post-surgery inflammation (for example anterior chamber reaction or corneal edema) that might influence image quality. For example, we assume that the significantly lower retinal thickness observed 3 days after XEN (p = 0.018) compared to TE treated patients, is due to the significantly worse image quality (p = 0.028) during that period. For our study, we tried to include all intervals that seemed significant for the investigation but as aforementioned, we could not detect significant changes or specific trends in VAD at any follow-up interval.

Regarding the FAZ results, Shoji et al.^31^ and Park et al.^32^ were able to detect a significant change and reduction 3 months after glaucoma surgery, whereas we could not. This might be linked to a lack of accuracy of our method. Shoji et al.^31^ used the same OCT-A but different external software programs on their study that allowed for magnified view and specific color coding making the tracing potentially more appropriate to detect changes in the FAZ. On the other hand, our results of the lack of change in FAZ match to our results of VAD. In contrast to In et al.^24^, who found a significantly increased VAD after TE treatment, and Park et al.^32^, who showed an increase in VAD in the optic disc and a decrease of FAZ after glaucoma surgery, our patients had a preoperative, moderately IOP elevated glaucoma (about 20 mmHg). The IOP amplitude was significantly lower compared to their study where the IOP amplitude was high and IOP preoperatively was poorly regulated (about 30 mmHg)^24^. It is possible, that the effect of IOP reduction in preoperative moderately IOP controlled glaucoma, might not be as influential to changes in the VAD. This is additionally supported by our correlation analysis where none of the vessel complexes significantly correlate to changes in IOP. The influence of higher pre-surgery baseline IOP values has been discussed before and it is speculated that the “lower pressure range autoregulation can compensate the changes” in the VAD adequately as stated by Lommatzsch et al.^25^. Another potential influence might be initial severity of glaucoma as measured by, for example, the visual field MD. Miraftabi et al.^30^, who had patients with slightly more pronounced visual field loss as obtained from the higher baseline MD values, observed a significant increase in VAD after TE. It could be argued again that severity of glaucoma might influence the vascular architecture.

Kim et al.^21^ linked their improved diagnostic outcome of the vascular parameters more to the reduction of lamina cribrosa depth (LCD) rather than to the IOP reduction. Changes in LCD were not analyzed in this study.

Filtrating glaucoma surgery has become more varied in recent years and, as aforementioned, offers the best IOP lowering alternative. The XEN stent has a lower IOP lowering effect than the TE treatment. In addition, we find a slight decrease of the RNFL after 6 months for the XEN group. No significant increase in VAD could be detected, but VAD also did not decrease. This lets us deduct or at least assume that aside from lowering IOP, filtrating surgery might also stabilize vasculature architecture in glaucoma-affected eyes. In comparing the two procedures with one another, TE treated eyes showed significantly higher VAD values in the SVC, DVC and the optic disc after 6 months. These better results, and the more effective IOP reduction after TE treatment, could point out that a long-term sufficiently lowered IOP could potentially better stabilize the RNFL and VAD. The positive influence of the type of glaucoma surgery on VAD is probably a further reason aside from higher preoperative IOP levels for significant VAD differences after filtrating surgery as reported by In et al.^24^ and Park et al.^32^, where the majority of patients received trabeculectomy and only a small fraction different shunt implantation.

On the other hand, the preoperative RNFL was also higher in the TE group than in the XEN group without a significant difference. To get more information about the long-term effects after filtrating surgery, longer follow-up periods would be necessary.

Early hypotony, occurring in four patients in this study, also showed no significant influence on the VAD in the overall course.

Though analysis of VAD might not have been as indicative for detection of change in post-surgery vasculature architecture, our correlation analysis showed a significant correlation between the extent of glaucomatous damage, given in functional parameters, and VAD. More specifically, MD and RNFL significantly correlated with several layers of perfusion before and after surgery. Our findings go along with previous propositions made by Yarmohammadi et al.^4,33^ where the extent of glaucomatous damage could be associated to the microvascularization. OCT-A, in itself, might therefore be helpful in complementing and offering a wider range of glaucomatous damage. Specifically, through detection of early changes in microvascularization that could possibly be seen prior to irreversible RNFL or visual field loss. These changes, along with classical parameters like IOP, could offer an enhancement in the current management and potential screening of glaucoma patients.

The results of this study are assessable to a certain extent. Vascular systemic diseases such as diabetes and high blood pressure need be excluded in future studies. On the other hand, these comorbidities are very common in glaucoma patients too. Thus we show real life data after glaucoma surgery. Intra-statistical analysis of patients, as also performed in our study, allows a reduction in that bias compared to inter-statistical analysis. The influence and impact of image quality on the results should also be considered as it is known that this can significantly influence the results^34^. We have tried to minimize chance of influencing by setting a minimum to the signal strength index (not below 20). Czakó et al.^34^ indicate that the repeatability of OCTA parameters increased along with increasing image quality. Additionally, we also did not find a significant difference in image quality between the two groups expect of 3 days after surgery as discussed before. The sample size of the study might also influence the statistical significance of the results. Due to the number of patients and the ratio of TE to XEN patients, it should be pointed out that a lack of statistically significant difference does not directly imply that both treatment groups are the same or similar in terms of the results. In addition, analysis and anatomical definition of perfusion layer slightly varies depending on the OCT-A device used and is therefore defined differently in other studies. A more standardized definition and classification of perfusion layer is necessary in the future, ideally with a standardized method for VAD analysis. However, we find significant correlations comparable to results in current literature.

## Conclusion

The current study situation regarding microvasculature after filtrating surgery remains ambivalent. Our study’s findings can imply that VAD does not significantly improve after filtrating surgery in preoperative moderately IOP elevated eyes, but IOP lowering by filtrating surgery could potentially stabilize the vascular architecture. VAD seems to correlate with the extent of glaucomatous damage, which could have potential in diagnosing and caring for glaucoma patients. Future studies should have a more standardized approach to evaluate VAD and perfusion layers so that findings are more comparable.

## Data Availability

We have full access to all the data in the study and take responsibility for the integrity of the data and the accuracy of the data analysis. The datasets used and/or analyzed during the current study are available from the corresponding author on reasonable request.
